# Supplementary material for: Life Stages and Phylogenetic Position of the New Scale-Mite of the Genus Neopterygosoma (Acariformes: Pterygosomatidae) from Robert’s Tree Iguana
Source: Animals (Basel). 2023 Sep 4;13(17):2809. doi: 10.3390/ani13172809 (PMC10487052; doi:10.3390/ani13172809)
Supplement: Supplementary file 1 [file animals-13-02809-s001.zip › Supplement1 List of morph char.pdf]

Supplement 1. List of morphological characters and character states used in the analyses (based on females only).

1. *Gnathosoma situated*: **(0)** apically; **(1)** displaced on dorsal side; **(2)** dispaced on ventral side.
2. *Fixed cheliceral digit*: **(0)** spinous (smooth or with tines); **(1)** rounded (greatly reduced).
3. *Movable cheliceral digit*: **(0)** without basal spur; **(1)** with basal spur.
4. *Cheliceral shaft (narrow part)*: **(0)** longer than swollen cheliceral base; **(1)** equal or subequal with swollen cheliceral base; **(2)** shorter than swollen cheliceral base.
5. *Setal form of dorsal seta (dF) of palpal femur*: **(0)** tapered; **(1)** spur-like.
6. *Palp setae dF*: **(0)** smooth; **(1)** serrate.
7. *Serration of setae dF*: **(0)** serrate only distally; **(1)** serrate on all length.
8. *Shape of setae dF*: **(0)** tapered; **(1)** spur-like.
9. *Length of setae dF*: **(0)** shorter than *dG*; **(1)** equal or subequal with *dG*; **(2)** longer than *dG*.
10. *Palp setae dG*: **(0)** smooth; **(1)** serrate.
11. *Serration of setae dG*: **(0)** serrate only on distal half; **(1)** serrate on all length.
12. *Lateral seta l' on palpal tibia (l'Ti)*: **(0)** smooth; **(1)** with barely discernible serration.
13. *Palpal tarsus*: **(0)** fully articulated from palpal tibia; **(1)** partially or completely fused with palpal tibia.
14. *Seta sul of palpal tarusus*: **(0)** present; **(1)** absent.
15. *Eupathidion ul' of palpal tarsus*: **(0)** present ; **(1)** absent.
16. *Eupathidion ul'' of palpal tarsus*: **(0)** present; **(1)** absent.
17. *Solenidion ω1 on palpal tarsus*: **(0)** present; **(1)** absent.
18. *Length of setae n*: **(0)** very short (35–45 long); **(1)** short (55–65 long); **(2)** average (70–90 long); **(3)** long (100–110 long); **(4)** very long (≥125 long).
19. *Subcapitular setae n*: **(0)** smooth; **(1)** serrate.
20. *Serration of setae n*: **(0)** serrate only at tip; **(1)** with barely discernible serration on all length; **(2)** slightly serrate on all length; **(3)** densely serrate on all length.
21. *Peritremes*: **(0)** with barely visible chambers; **(1)** with clearly visible chambers.
22. *Number of peritremal chambers*: **(0)** less than 6; **(1)** 19–22; **(2)** 23–26; **(3)** >27.
23. *Free peritremal branch*: **(0)** present; **(1)** absent.
24. *Length of free peritremal branch (in µm)*: **(0)** short (<10 long); **(1)** avarage (40 long); **(2)** long (>55).
25. *Shape of idiosoma*: **(0)** longer than wide; **(1)** wider than long; **(2)** circular (almost as long as wide).
26. *Eyes situated*: **(0)** dorsally, outside propodonotal shield in lateral parts of idiosoma; **(1)** dorsally on propodonotal shield; **(2)** on ventral side between antero-lateral setae.
27. *Idiosomal hypertrichy*: **(0)** absent; **(1)** present.
28. *Numerous setae in mid-dorsal part of idiosoma*: **(0)** present; **(1)** absent.
29. *Numerous antero-median dorsal setae*: **(0)** grouped into mid-dorsal cluster; **(1)** not grouped into cluster.
30. *Setae of mid-dorsal cluster*: **(0)** increase in length from anterior to posterior part of the cluster; **(1)** subequal in length.
31. *Setae in mid-dorsal cluster grouped*: **(0)** in two distinct rows (small clusters); **(1)** in several rows (one large cluster).

32. *Length of setae in mid-dorsal cluster*: **(0)** short (<30 μm); **(1)** long (55–95 μm).
33. *Number of setae in mid- dorsal cluser*: **(0)** 10–20 ; **(1)** 40–50; **(2)** 60–70; **(3)** 80–90; **(4)** > 110.
34. *Antero-median setae*: **(0)** plumose; **(1)** with minute serration.
35. *Number of antero-lateral sete*: **(0)** 210–230; **(1)** 150–209; **(2)** 149–90; **(3)** <90.
36. *Number of dorso-median setae dm*: **(0)** >15 pairs of setae; **(1)** 1–5 pairs of setae.
37. *One much longer seta situated between antero-lateral setae*: **(0)** present; **(1)** absent.
38. *Setae vm1*: **(0)** smooth; **(1)** serrate.
39. *Serrate setae vm1*: **(0)** with barely discernible serration; **(1)** slightly serrate; **(2)** densely serrate.
40. *Number of ventro-median setae vm*: **(0)** over 10 pairs; **(1)** only 1-3 setae on each side.
41. *Setae vm*: **(0)** densely serrate; **(1)** slightly serrate.
42. *Setae on postero-lateral part of idiosomal dorsum*: **(0)** subequal with the remaining dorsal setae situated in anterior half of idiosoma; **(1)** much longer than the setae in anterior half of idiosoma.
43. *Setal form of long peripheral setae*: **(0)** tapered; **(1)** slightly apically expanded at tip.
44. *Peripheral setae*: **(0)** much longer than dorsal setae situated medially and laterally; **(1)** subequal with median and lateral setae on idiosomal dorsum.
45. *Peripheral setae situated dorsally and ventrally*: **(0)** much longer than the setae situated in anterior half of idiosomal dorsum; **(1)** subequal with setae situated in anterior half of idiosomal dorsum.
46. *Genital slit situated*: **(0)** ventrally; **(1)** dorsally or terminally.
47. *Genital setae*: **(0)** smooth; **(1)** serrate.
48. *Serration of genital setae*: **(0)** with barely discernible serration; **(1)** clearly serrate.
49. *Genital setae g2*: **(0)** absent; **(1)** present.
50. *Genital setae g3*: **(0)** absent; **(1)** present.
51. *Genital setae g4*: **(0)** absent; **(1)** present.
52. *Genital setae g5*: **(0)** absent; **(1)** present.
53. *Pseudoanal setae ps1*: **(0)** present; **(1)** absent.
54. *Setal form of setae ps1*: **(0)** tapered; **(1)** slightly apically expanded at tip.
55. *Pseudoanal setae ps2*: **(0)** present; **(1)** absent.
56. *Setal form of setae ps2*: **(0)** tapered; **(1)** slightly apically expanded.
57. *Pseudoanal setae ps3*: **(0)** present; **(1)** absent.
58. *Setal form of setae ps3*: **(0)** tapered; **(1)** slightly apically expanded.
59. *Pseudoanal setae ps4*: **(0)** absent; **(1)** present.
60. *Pseudoanal setae ps5*: **(0)** absent; **(1)** present.
61. *Length of legs II*: **(0)** considerably shorter than legs I; **(1)** slightly shorter than legs II; **(2)** subequal with legs I.
62. *Legs III and IV*: **(0)** directed posteriorly; **(1)** directed anteriorly.
63. *Length of coxae I*: **(0)** subequal to coxae II; **(1)** longer than coxae II.
64. *Coxal setae 1c*: **(0)** absent; **(1)** present.
65. *Setae 1a situated*: **(0)** on coxal plate; **(1)** ouside coxal plate.
66. *Coxal field II*: **(0)** with a thick or fine line of separation between coxa III and IV; **(1)** without a line of separation between coxa III and IV.
67. *Coxal setae 2a*: **(0)** present; **(1)** absent.
68. *Setal form of coxal setae 2b*: **(0)** simple; **(1)** spur-like.
69. *Shape of setae 3a:* **(0)** tapered; **(1)** spur-like.

70. *Coxal setae 3a*: **(0)** smooth; **(1)** serrate.
71. *Serration of coxal setae 3a*: **(0)** slightly serrate or with barely discernible serration; **(1)** densely serrate.
72. *Setae 3a situated*: **(0)** on coxal plate; **(1)** outside coxal plate.
73. *Coxal setae 3c*: **(0)** present; **(1)** absent.
74. *Coxal setae 3d*: **(0)** present; **(1)** absent.
75. *Coxal setae 4a*: **(0)** present; **(1)** absent.
76. *Setal form of setae 4a*: **(0)** simple; **(1)** spur-like.
77. *Coxal setae 4b*: **(0)** absent; **(1)** present.
78. *Coxal setae 4c*: **(0)** present; **(1)** absent.
79. *Setal form of vTrI*: **(0)** tapered; **(1)** spur-like.
80. *Setal form of vTrII*: **(0)** tapered; **(1)** spur-like.
81. *Setal form of vTrIV*: **(0)** tapered; **(1)** spur-like.
82. *Setae v'TrI-IV*: **(0)** serrate; **(1)** smooth.
83. *Serration of seta v'TrI-IV*: **(0)** slightly serrate; **(1)** densely serrate.
84. *Seta ld'FI*: **(0)** present; **(1)** absent.
85. *Seta lv' on femur I*: **(0)** present; **(1)** absent.
86. *Seta dFII*: **(0)** present; **(1)** absent.
87. *Seta ld" on femur II*: **(0)** present; **(1)** absent.
88. *Seta lv" on femur II*: **(0)** absent; **(1)** present.
89. *Seta d on femur III*: **(0)** present; **(1)** absent.
90. *Seta ld' on femur III*: **(0)** present; **(1)** absent.
91. *Seta ld" on femur III*: **(0)** absent; **(1)** present.
92. *Seta lv' on femur III*: **(0)** absent; **(1)** present.
93. *Seta v on femur III*: **(0)** present; **(1)** absent.
94. *Seta d on femur IV*: **(0)** present; **(1)** absent.
95. *Seta ld' on femur IV*: **(0)** present; **(1)** absent.
96. *Seta ld" on femur IV*: **(0)** absent; **(1)** present.
97. *Seta lv' on femur IV*: **(0)** absent; **(1)** present.
98. *Seta v on femur IV*: **(0)** present; **(1)** absent.
99. *Seta d on genu I*: **(0)** present; **(1)** absent.
100. *Seta ld" on genu I*: **(0)** present; **(1)** absent.
101. *Seta v" on genu I*: **(0)** present; **(1)** absent.
102. *Seta d on genu II*: **(0)** present; **(1)** absent.
103. *Seta ld' on genu II*: **(0)** present; **(1)** absent.
104. *Seta ld" on genu II*: **(0)** present; **(1)** absent.
105. *Seta v" on genu II*: **(0)** present; **(1)** absent.
106. *Seta d on genu III*: **(0)** present; **(1)** absent.
107. *Seta ld' on genu III*: **(0)** present; **(1)** absent.
108. *Seta lv' on genu III*: **(0)** present; **(1)** absent.
109. *Seta d on genu IV*: **(0)** present; **(1)** absent.
110. *Seta ld' on genu IV*: **(0)** present; **(1)** absent.
111. *Seta lv' on genu IV*: **(0)** present; **(1)** absent.
112. *Solenidion on tibia III*: **(0)** absent; **(1)** present.
113. *Solenidion $\omega 2$ on tarsi I*: **(0)** present; **(1)** absent.
114. *Length of fastigial seta ft*: **(0)** shorter than solenidion $\omega 1$; **(1)** longer than solenidion $\omega 1$.

115. *Seta tc" on tarsi II*: **(0)** serrate; **(1)** smooth.
116. *Solenidion w on tarsi III*: **(0)** present; **(1)** absent.
117. *Setae a' and a" of tarsi I*: **(0)** smooth; **(1)** serrate.
118. *Setae tc' and tc" of legs II–IV*: **(0)** smooth; **(1)** serrate.
119. *Serration of setae tc' and tc" of legs II–IV*: **(0)** with barely discernible serration; **(1)** slightly serrate; **(2)** clearly serrate .
120. *Setae vs' and vs' of legs IV*: **(0)** with barely discernible serration; **(1)** slightly serrate; **(2)** densely serrate; **(3)** thick and densely serrate.
