# Supplementary material for: Life Stages and Phylogenetic Position of the New Scale-Mite of the Genus Neopterygosoma (Acariformes: Pterygosomatidae) from Robert’s Tree Iguana
Source: Animals (Basel). 2023 Sep 4;13(17):2809. doi: 10.3390/ani13172809 (PMC10487052; doi:10.3390/ani13172809)
Supplement: Supplementary file 1 [file animals-13-02809-s001.zip › Table S1.pdf]

Table S1. Matrix of morphological characters used in phylogenetical analysis (Abbreviation of species names as in Table 1)

**Taxa**	**1**	**2**	**3**	**4**	**5**	**6**	**7**	**8**	**9**	**10**	**11**	**12**	**13**	**14**	**15**	**16**	**17**	**18**	**19**	**20**	**21**	**22**	**23**	**24**	**25**	**26**	**27**	**28**	**29**	**30**	**31**	**32**	**33**	**34**	**35**	**36**	**37**	**38**	**39**	**40**
Pim. pod.	0	0	0	0	0	0	-	0	0	0	-	0	0	0	0	0	0	1	0	-	0	0	0	0	0	0	0	-	-	-	-	-	-	-	-	-	-	-	-	-
Gec. nit.	0	1	0	0	0	1	1	0	2	1	1	1	0	1	0	1	0	2	1	0	1	1	1	-	1	-	1	0	0	1	0	1	0	1	3	0	1	0	-	0
Gec. ger.	1	0	1	0	1	1	0	1	2	0	-	0	1	1	1	1	1	1	0	-	0	0	1	-	1	1	1	0	1	-	-	-	-	1	3	-	1	-	-	-
Gec. hir.	0	0	1	1	0	1	1	0	0	1	1	0	1	1	1	1	1	0	1	2	0	0	1	-	2	1	1	0	1	-	-	-	-	1	3	-	1	-	-	-
Neo. pat.	0	0	0	0	0	1	1	0	2	1	1	0	0	1	0	1	0	0	1	2	1	2+3	0	0	2	2	1	1	0	1	1	0	1	0	1	0	1	1	2	0
Neo. for.	0	1	0	1	0	1	1	0	0	1	1	0	0	1	0	1	0	2	0	-	1	3	0	1	1	2	1	1	0	1	1	0	4	0	0	0	0	1	2	0
Neo. ova.	1	1	0	0	0	1	1	0	0	1	0	0	0	1	0	1	0	4	0	-	1	3	1	-	1	2	1	1	0	1	1	0	3	0	1	0	1	1	2	0
Neo. lig.	0	1	0	0	0	1	1	0	2	1	1	0	0	1	0	1	0	3	0	-	1	3	0	2	1	2	1	1	0	0	1	0	3	0	1	0	0	1	1	0
Neo. lev.	0	1	0	1	0	1	1	0	2	1	1	0	0	1	0	1	0	2	0	-	1	2	0	1	1	2	1	1	0	1	1	0	3	0	0	0	0	1	0	0
Neo. chil.	0	1	0	0	0	1	1	0	2	1	1	0	0	1	0	1	0	1	1	1	1	3	0	1	1	2	1	1	0	0	1	0	2	0	1	0	0	1	0	0
Neo. schr.	0	0	0	2	0	1	1	0	1	1	0	0	0	1	0	1	0	4	1	1	1	1	0	0	1	2	1	1	0	1	1	0	2	0	0	0	1	0	2	0
Neo. cya.	0	0	0	2	0	1	0	0	2	1	0	1	0	1	0	1	0	2	1	3	0	2	0+	0	1	2	1	1	0	1	1	0	2	0	0	0	0	0	-	0
Neo. rob.	0	0	0	1	0	1	1	0	2	1	0	1	0	1	0	1	0	2	0	-	0	1	1	-	1	2	1	1	0	1	1	0	2	0	2	1	1	1	2	1

**Taxa**	**41**	**42**	**43**	**44**	**45**	**46**	**47**	**48**	**49**	**50**	**51**	**52**	**53**	**54**	**55**	**56**	**57**	**58**	**59**	**60**	**61**	**62**	**63**	**64**	**65**	**66**	**67**	**68**	**69**	**70**	**71**	**72**	**73**	**74**	**75**	**76**	**77**	**78**	**79**	**80**

Pim. pod.	-	-	-	-	-	0	1	0	0	0	0	0	0	0	0	0	0	0	0	0	0	0	0	0	-	0	0	0	0	0	-	-	0	0	0	0	0	0	0	0
Gec. nit.	0	0	-	1	1	0	0	-	1	0	0	0	0	0	0	0	0	0	1	1	0	1	0	0	0	1	0	0	0	0	-	0	0	0	0	0	0	1	0	0
Gec. ger.	-	1	1	1	0	0	0	-	1	1	0	0	1	-	1	-	1	-	0	0	2	1	0	0	-	1	0	1	1	1	1	-	0	0	0	1	1	0	1	1
Gec. hir.	-	0	-	1	1	0	0	-	1	1	1	0	1	-	1	-	1	-	0	0	2	1	0	0	-	1	0	1	1	1	1	-	1	1	0	1	0	1	0	0
Neo. pat.	0	0	-	1	1	0	1	0	0	0	0	0	0	0	0	0	0	0	1	1	2	0	1	0	0	1	1	0	0	1	0	0	0	0	1	-	0	1	0	0
Neo. for.	0	1	1	0	0	0	1	1	0	0	0	0	0	0	0	0	0	0	0	0	1	0	1	0	0	1	1	0	0	1	0	0	0	0	1	-	0	1	0	0
Neo. ova.	0	1	1	0	0	1	1	1	0	0	0	0	0	0	0	0	0	0	1	1	0	0	1	1	0	1	1	0	0	1	0	1	0	0	1	-	0	1	0	0
Neo. lig.	0	1	1	0	0	0	1	1	0	0	0	0	0	0	0	0	0	0	1	1	0	0	1	0	0	1	1	0	0	1	0	0	0	0	1	-	0	1	0	0
Neo. lev.	0	1	0	0	0	1	1	1	0	0	0	0	0	0	0	0	0	0	1	1	0	0	1	0	0+	1	1	0	0	0	-	1	0	0	1	-	0	1	0	0
Neo. chil.	1	1	0	0	0	0	1	1	0	0	0	0	0	0	0	0	0	0	1	1	0	0	1	0	1	1	1	0	0	0	-	1	0	0	1	-	0	1	0	0
Neo. schr.	0	1	1	0	0	0	1	0	0	0	0	0	0	0	0	0	0	0	1	0	0	0	1	0	1	1	1	0	0	0	-	1	0	0	1	-	0	1	0	0
Neo. cya.	0	1	1	0	0	0	0	-	0	0	0	0	0	0	0	0	0	0	1	1	1	0	1	0	0	1	1	0	0	1	0	0	0	0	1	-	0	1	0	0
Neo. rob.	0	1	1	0	0	0	1	1	1	1	1	1	0	1	0	1	0	1	1	1	1	0	1	0	0+	1	1	0	0	0	-	1	0	0	1	-	0	1	0	0
**Taxa**	**81**	**82**	**83**	**84**	**85**	**86**	**87**	**88**	**89**	**90**	**91**	**92**	**93**	**94**	**95**	**96**	**97**	**98**	**99**	**100**	**101**	**102**	**103**	**104**	**105**	**106**	**107**	**108**	**109**	**110**	**111**	**112**	**113**	**114**	**115**	**116**	**117**	**118**	**119**	**120**

Pim. pod.	0	0	0	0	0	0	0	0	0	0	0	0	0	0	0	0	0	0	0	0	0	0	0	0	0	0	0	0	0	0	0	0	0	0	0	0	0	0	-	-
Gec. nit.	0	0	1	0	0	0	0	0	0	0	0	1	1	0	0	0	1	1	0	0	0	0	0	0	0	0	0	1	0	1	0	0	1	1	0	1	1	1	2	3
Gec. ger.	1	0	1	1	1	1	0	0	1	1	1	0	0	1	1	1	0	0	0	1	1	1	1	1	1	1	1	1	1	1	1	0	1	0	0	1	1	0	-	1
Gec. hir.	0	0	1	1	1	0	1	0	0	1	1	0	1	0	1	1	0	1	1	1	1	1	1	1	1	1	1	1	1	1	0	1	1	0	0	1	1	?	?	?
Neo. pat.	0	0	0	0	0	0	0	0	0	0	0	0	0	0	1	0	0	0	0	0	0	0	0	0	1	0	0	1	0	0	1	0	1	1	1	1	0	0	-	0
Neo. for.	0	1	-	0	0	0	1	0	0	0	0	0	0	0	1	0	0	0	0	0	1	0	0	0	1	0	0	1	0	0	0	0	1	1	0	1	1	1	2	0
Neo. ova.	0	0	0	0	0	0	0	1	0	0	0	0	0	0	0	0	0	0	0	0	0	0	0	0	1	0	0	1	0	0	0	0	1	1	0	1	1	1	2	2
Neo. lig.	0	0	0	0	0	0	0	1	0	0	0	0	0	0	0	0	0	0	0	0	0/1	0	0	0	1	0	0	1	0	0	0	0	1	1	0	1	0	1	2	2
Neo. lev.	0	0	1	0	0	0	0	1	0	0	0	0	0	0	0	0	0	0	0	0	0	0	0	0	1	0	0	1	0	0	0	0	1	1	0	1	1	1	2	0
Neo. chil.	0	0	1	0	0	0	0	0	0	0	0	0	0	0	0	0	0	0	0	0	0	0	0	0	1	0	0	1	0	0	0	0	1	1	0	1	1	1	2	1
Neo. schr.	0	1	-	0	0	0	0	0	0	0	0	0	0	0	0	0	0	0	0	0	0	0	0	0	1	0	0	1	0	0	0	0	1	1	0	1	0	1	2	1
Neo. cya.	0	0	0+	0	0	0	0	1	0	0	0	0	0	0	0	0	0	0	0	0	0	0	0	0	1	0	0	1	0	0	0	0	1	1	0	1	0	0	-	1
Neo. rob.	0	0	0	0	0	0	0	0	0	0	0	0	0	0	1	0	0	0	0	0	0	0	0	0	1	0	0	1	0	0	1	0	1	1	0	1	0	0+1	0	0
